# Size-Energy Relationships in Ecological Communities

**DOI:** 10.1371/journal.pone.0068657

**Published:** 2013-08-07

**Authors:** Brent J. Sewall, Amy L. Freestone, Joseph E. Hawes, Ernest Andriamanarina

**Affiliations:** 1 Department of Biology, Temple University, Philadelphia, Pennsylvania, United States of America; 2 Department of Wildlife, Fish, and Conservation Biology, University of California Davis, Davis, California, United States of America; 3 School of Environmental Sciences, University of East Anglia, Norwich Research Park, Norwich, United Kingdom; 4 Département des Sciences de la Nature et de l'Environnement, Université d'Antsiranana, Antsiranana, Madagascar; Utah State University, United States of America

## Abstract

Hypotheses that relate body size to energy use are of particular interest in community ecology and macroecology because of their potential to facilitate quantitative predictions about species interactions and to clarify complex ecological patterns. One prominent size-energy hypothesis, the energetic equivalence hypothesis, proposes that energy use from shared, limiting resources by populations or size classes of foragers will be independent of body size. Alternative hypotheses propose that energy use will increase with body size, decrease with body size, or peak at an intermediate body size. Despite extensive study, however, size-energy hypotheses remain controversial, due to a lack of directly-measured data on energy use, a tendency to confound distinct scaling relationships, and insufficient attention to the ecological contexts in which predicted relationships are likely to occur. Our goal, therefore, was to directly evaluate size-energy hypotheses while clarifying how results would differ with alternate methods and assumptions. We comprehensively tested size-energy hypotheses in a vertebrate frugivore guild in a tropical forest in Madagascar. Our test of size-energy hypotheses, which is the first to examine energy intake directly, was consistent with the energetic equivalence hypothesis. This finding corresponds with predictions of metabolic theory and models of energy distribution in ecological communities, which imply that body size does not confer an advantage in competition for energy among populations or size classes of foragers. This result was robust to different assumptions about energy regulation. Our results from direct energy measurement, however, contrasted with those obtained with conventional methods of indirect inference from size-density relationships, suggesting that size-density relationships do not provide an appropriate proxy for size-energy relationships as has commonly been assumed. Our research also provides insights into mechanisms underlying local size-energy relationships and has important implications for predicting species interactions and for understanding the structure and dynamics of ecological communities.

## Introduction

Body size has strong, widespread, and predictable relationships to organisms' physiological traits and life history characteristics [1], [2], and has therefore been proposed as a primary driver of diverse patterns in community ecology and macroecology [2], [3]. Relationships between body size and energy use in particular have long intrigued ecologists [4], [5], because they promise to reveal an underlying order in highly complex ecological systems, and to predict quantitative patterns of species interactions from local to global spatial scales.

One prominent hypothesis relating body size to energy use was proposed by Damuth, who focused on three variables of interest – the density (*D*) of a population, the mean metabolic (respiration) rate (*R*) of individuals of that population, and the total amount of energy used by all individuals of that population, or population energy use (*PEU*) – as well as the relationships of each of these three variables to body size, as indicated by body mass (*M*). In a widely-cited paper [6], he suggested that population energy use could be estimated as the product of population density and mean metabolic rate (i.e., *PEU* = *D* * *R*). To relate these variables to body size, he compiled a global dataset of population densities of mammalian herbivores from published accounts, and found that population density (*D*) scaled globally with body mass (*M*) to the power of −0.75 [6]. Noting that metabolic rate (*R*) was already understood to scale with body mass (*M*) to the power of +0.75 [1], Damuth then hypothesized that the total energy used by a population should generally be independent of body size [6], because *PEU* = *D* * *R*∝*M* ^−0.75^ * *M* ^0.75^ = *M* ^0^. This finding, later called the ‘energetic equivalence rule’ [7], has been highly influential, because it suggests that underlying the complexities of ecological communities, populations of animals of all body sizes are on equal footing in the competition for energy (i.e., there is no optimal body size where energy use is maximized) [6]. The energetic equivalence rule has also garnered widespread attention since it provides a quantifiable prediction for a point of great interest in ecology – the distribution of energy among populations in a community – on the basis of a relatively easily-measured variable, body size. This energetic equivalence rule, and its emphasis on three-quarter-power scaling with body mass, has provided the theoretical foundation for explaining a range of ecological patterns, from population growth [8] to community structure [9] to global biodiversity patterns [10].

Later authors [5], [11], [12] expanded on this idea, noting that population energy use would also be independent of body size wherever both scaling exponents were exactly inversely correlated. Thus, if *D*∝*M^a^*, *R*∝*M^b^*, and *PEU*∝*M^c^*, then *M^a^* * *M^b^* = *M^c^* and population energy use will be independent of body size whenever *c* = 0. This will occur whenever *−a* = *b*, even if *−a* and *b* are not equal to 0.75 in the system being studied. Thus, the ‘energetic equivalence rule’ is a special case (where *a* = −0.75, *b* = +0.75) of a broader hypothesis known as ‘energetic equivalence’ [5], where *−a* = *b*. This energetic equivalence hypothesis predicts that inverse scaling relationships of population density and metabolic rate are widespread, and that population energy use by different species is broadly independent of body size in ecological communities [13].

While the energetic equivalence hypothesis has been supported in some studies [14], [15], this hypothesis is only one possible expectation for energy use within ecological communities [4]. Other studies have proposed and provided evidence in support of alternative hypotheses: that energy use increases with body size (e.g., [16]), decreases with body size (e.g., [12]), or peaks at an intermediate body size (e.g., [17]). Thus, despite intense interest in size-energy hypotheses, and despite their potential importance for understanding community structure and dynamics, past studies have produced conflicting results and the influence of body size on energy use in ecological communities remains unclear.

In addition, size-energy hypotheses have been controversial because they are often disconnected from the proposed mechanism underlying these hypotheses – namely, that size-energy relationships result from energetic tradeoffs due to resource competition among organisms of different body sizes in a community [2], [5], [6] but see [18]. This mechanism implies that size-energy hypotheses apply only in certain ecological contexts – namely those where all species can directly compete for shared, limiting resources. Previous studies of size-energy hypotheses, however, have tended to examine large spatial scales by compiling data from many different communities around the globe (e.g., [6]), though species selected for analysis from separate communities cannot plausibly compete [19]. Further, those studies that have examined the local scale have not controlled for resource availability (e.g., [20]), though the use of different resources implies a lack of energetic tradeoffs due to competition [2], [20]. Inferences about size-energy hypotheses from large spatial scale or multi-resource studies should therefore be interpreted carefully.

Size-energy hypotheses, and past tests of these hypotheses, have also been controversial on methodological grounds, in part due to a tendency to confound distinct scaling relationships in two primary ways. First, all previous studies have indirectly used size-density relationships, rather than size-energy relationships, to test size-energy hypotheses (e.g., [11], [21], [22]). This method (hereafter, the ‘indirect method’) has spread because in practice it is easier to measure the density scaling relationship (i.e., the exponent *a*) than the energy scaling relationship (*c*) in a community. In such studies, the exponent of the metabolic scaling relationship (*b*) is assumed to be a universal constant (+0.75) (e.g., [6], [14], [15], [23]), or is taken from published metabolic scaling relationships when available (e.g., [24]), such that *c* can be inferred directly from *a* via the equation *M^a^* * *M^b^* = *M^c^*, or *a*+*b* = *c*. However, recent study suggests that metabolic scaling exponents are highly variable [25], and so a specific exponent cannot be assumed for all systems (i.e., *b* is not constant across all systems, and thus *a* is insufficient to determine *c*). This approach also suffers from a lack of data on species-specific metabolic scaling exponents and is subject to the hidden non-linearity, propagation of error variances, and inherent imprecision of multiplying allometric relationships [21], [26]. This practice is also circular, since in the analysis, body size serves both as the predictor variable (*M*) and as a means to calculate the response variable (*PEU*); results therefore will tend to be biased to suggest a positive correlation. As a result, although size-density relationships are interesting in their own right [5], and can provide insight into mechanisms underlying energy use, their utility for estimating the form of the size-energy relationships that are the focus of size-energy hypotheses or for providing a robust test of these hypotheses remains unclear [19]. Direct measurement of energy use (hereafter, the ‘direct method’) could avoid these problems, but measurement of energy use by all species in a community is technically challenging [4], and no test of size-energy hypotheses using direct measurements of energy use has previously been attempted [27].

Second, two distinct approaches to evaluating size-energy hypotheses have emerged on the basis of the underlying assumptions for how energy use is regulated in ecological communities. Competition for energy implies zero-sum dynamics [13], [28], where limited resources are allocated to individual organisms in the community [29] and thus increases in energy consumed from a shared, limiting resource by one individual are necessarily offset by equal and opposite decreases in energy consumed by other individuals. Authors of studies on terrestrial animals have generally assumed that such competition for energy is regulated by competition among species populations in a community (e.g., [6]), while authors of studies on trees and marine systems have generally assumed that energy use is regulated by competition among size classes in a community (e.g., [30], [31]). For the former assumption, population energy use (*PEU*) is used to evaluate the size-energy relationship, whereas for the latter assumption, a related but distinct variable – size class energy use (*SCEU*) – is used. Unlike *PEU*, which is calculated as the sum of the energy used by all individuals in a species population, *SCEU* is calculated as the sum of the energy used by all individuals in a given size class, regardless of species identity. Because size classes may vary in the number of species they contain, and individuals of a single species may be distributed among multiple size classes, tests of size-energy hypotheses using each approach may provide differing results for the same community [32]. Despite these differences, reviews of size-energy hypotheses have typically not distinguished between the population and size-class approaches, leading to confusion in the literature [5]. Comparison of the two approaches in the same system could clarify underlying assumptions of how energy use is regulated in communities. Such comparisons have been completed for size-density relationships in multi-trophic communities [32] (see also [5], [33] for discussion), but not for size-energy relationships.

Our goal in this project was to directly evaluate size-energy hypotheses while clarifying how results would differ with alternate methods and assumptions. We had three objectives in support of this goal. First, we sought to complete the first analyses of size-energy hypotheses to directly measure how energy use scales with body size. Specifically, we evaluated whether energy use is independent of body size, increases with body size, decreases with body size, or peaks at an intermediate body size. Second, we sought to evaluate the influence of indirect versus direct methods on conclusions about size-energy hypotheses. Specifically, we compared results of our novel direct method with those from the indirect method conventionally used to test size-energy hypotheses. Third, we sought to determine the influence of assumptions about energy regulation on conclusions about size-energy hypotheses. Specifically, we compared results obtained using the population and size-class approaches. We addressed these objectives by examining scaling relationships among a guild of vertebrate frugivores as they foraged on a common set of shared, limiting resources in a tropical forest in Madagascar.

## Materials and Methods

### Ethics statement

This study was conducted at all times in strict accordance with the animal welfare protocols of the University of California, Davis and with the laws of the participating countries. Animal research was non-manipulative and completely observational, and solely examined wild animals in their natural habitat. The study was conducted on protected land owned by the Madagascar government and managed by Madagascar National Parks, and was approved by the Madagascar Ministère de l'Environnement, Direction Générale des Eaux et Forêts, under permits N° 143/MINEV.EF/SG/DGEF/DPB/SCBLF/RECH, N° 225/06/MINEV.EF/SG/DGEF/DPB/SCBLF/RECH, N° 024N-EV01/MG06, and N° 308N-EV11/MG06.

### Study system

We focused on frugivores, or fruit eating animals, due to their central ecological importance for the maintenance and regeneration of tropical forests [34], [35]. Specifically, we studied the vertebrate frugivore guild of Ankarana National Park, near Mahamasina in northern Madagascar, within 175 ha of semi-evergreen primary forest. This region undergoes two distinct seasons: a short wet season from January–April when almost all of the ∼1890 mm of annual rainfall occurs, and a longer dry season during the remaining months ([36]; pers. obs.). The study period was the end of the dry season (October–December 2005 and October 2006-early January 2007), at a time when available fruit is in limited supply and competition for shared resources is expected to be greatest. Frugivores are abundant and easily observable within the park, where they directly compete for clearly identifiable resources – primarily fruit from the canopies of fruiting trees. The frugivore guild comprised five bird, five primate (lemur), and two fruit bat species, a taxonomically diverse fauna with distinct foraging behaviors and body sizes spanning two orders of magnitude (Table 1).

**Table 1:** Vertebrate frugivore guild of Ankarana National Park, Madagascar, with taxa, activity, mass, and size class.

Species	English name	Taxon	Activity	Mass (g)^a^	Size class
*Saroglossa aurata*	Madagascar Starling	Bird	Diurnal	40	A
*Hypsipetes madagascariensis*	Madagascar Bulbul	Bird	Diurnal	43.5	A
*Microcebus tavaratra* b ^,^ c	northern rufous mouse lemur	Lemur	Nocturnal	64.5	B
*Rousettus madagascariensis*	Madagascar rousette	Bat	Nocturnal	65	B
*Cheirogaleus medius* c	fat-tailed dwarf lemur	Lemur	Nocturnal	198	C
*Treron australis*	Madagascar Green Pigeon	Bird	Diurnal	236	C
*Coracopsis nigra*	Lesser Vasa Parrot	Bird	Diurnal	254	D
*Eidolon dupreanum*	Madagascar straw-colored fruit bat	Bat	Nocturnal	295	D
*Phaner electromontis*	Amber Mountain fork-marked lemur	Lemur	Nocturnal	425	E
*Coracopsis vasa*	Greater Vasa Parrot	Bird	Diurnal	530	E
*Eulemur coronatus*	crowned lemur	Lemur	Cathemeral	1450	F
*Eulemur sanfordi*	Sanford's brown lemur	Lemur	Cathemeral	2150	G

a Based on the midpoint of the body mass range of adults of each species [72], [73], [74].

b We considered all *Microcebus* observations at Ankarana to be of *M. tavaratra* on the basis of recent taxonomic analyses [75].

c These or related species may enter torpor seasonally at other sites [73], but we observed them foraging throughout our study period.

### Indirect method for estimating size-energy relationships from size-density relationships

We first used the conventional indirect method for testing size-energy hypotheses, by measuring size-density relationships using frugivore population densities. For this portion of the study, we focused on frugivorous lemurs and bats. To determine lemur population densities, we established 12 standardized line transects of 500 m in length in primary forest, ≥100 m from each other and the forest edge. We walked each transect three times, during peak periods of frugivore foraging just after sunrise (05:00–08:00), and just before (15:00–18:00) and just after sunset (18:30–21:30). To control for animal behavior and detectability, we did not conduct observations during rain or wind, or during moonlit nights. We walked transects at 10 m per minute, searching both sides of the transect line for visual or auditory signs of lemurs. During nocturnal observations, we used two observers to ensure adequate coverage [37], and headlamps to facilitate lemur detections via direct sighting and eyeshine [38]. Upon detection, we identified each lemur to species and estimated perpendicular distance from the line to lemur individuals. To calculate densities of lemur populations from transect data, we determined a standard suite of detection functions separately for each species. We controlled for different effort during diurnal and nocturnal sampling with a multiplier [39], and we calculated densities of the two cathemeral species, *Eulemur coronatus* and *E. sanfordi*, solely from diurnal periods, when they were most active. We selected the best detection models following well-established methodology for distance sampling analyses [39], [40], and using Distance 6.0.2 software [41].

Since fruit bats were not easily detectable with distance sampling methods, we used roost count data to estimate their population densities. Because both species, *Rousettus madagascariensis* and *Eidolon dupreanum*, are capable of flying long distances, we used published estimates of their abundance at all roosts near Ankarana National Park [42], divided by the area of all primary and secondary forest in the same region [43].

We then converted densities to energy use with the conventional indirect approach, multiplying population density by individual field metabolic rate to determine energy use per unit area (kJ/(hr * km^2^)). As in past studies, we determined metabolic rates from allometric scaling relationships of similar species in the literature (equation 2 for eutherian mammals in [24]).

### Direct method for measuring size-energy relationships

We went beyond this conventional indirect method by developing a direct method for measuring the size-energy relationship. Size-energy hypotheses are typically formulated in relation to ‘energy use’ (e.g., population energy use, *PEU*), the amount of energy processed by all organisms of a population during metabolism, but this metric cannot easily be measured directly in the field. However, ‘energy intake’, the amount of metabolizable energy consumed by an organism, is both directly observable and closely correlated with energy use [44]. Energy intake is also directly linked to the proposed mechanism (of resource competition) that underlies size-energy hypotheses [5], [6], since energy consumed by one organism from a shared, limited resource in a habitat is not available to another organism, regardless of whether that consumed energy is actually processed. We therefore used energy intake as our metric by measuring fruit consumption during both diurnal and nocturnal foraging periods by the entire frugivore guild – including frugivorous birds, lemurs, and bats.

To determine the size-energy relationship, we measured energy intake by frugivore species while foraging at fig (*Ficus*, Moraceae) trees as a proxy for total energy intake. This approach assumes that figs were accessible to all frugivores and that the relative portion of energy intake at fig trees by frugivores was proportional to their total energy intake during the study period. The general importance of the frugivore-fig interaction, and several characteristics of our site – including strong preferences by frugivores for fig fruit, the abundance of fig fruit, scarcity of other resources, and evidence of rapid fig consumption – together suggest this assumption is justified in our study system. Specifically, the frugivore-fig interaction is critical to tropical forest communities as figs are widely distributed [45] and common in tropical forests [46]. Figs also play a keystone role in maintaining frugivore populations [47], [48], as they provide non-defended, easy-to-digest syconia (hereafter, fruit) year-round that is critical to a wide variety of frugivores during periods of fruit scarcity [48]. At our site, observations of frugivore foraging in fig trees and in the eight other most common non-fig species of fruiting trees during the dry season indicated strong preferences by all frugivore species for fig fruit, and no body-size trend among frugivores in relative preferences for fig fruit (KE Reuter, AA Gudiel, S Nieves, C Stanley, BJ Sewall, unpublished). During our study period in the late dry season, non-fig fruits were scarce: on the basis of fruit crop measurements at fig trees, and fruit counts at 10 randomly-placed transects of 50 m×2 m observed at two-week intervals, the three most common fig species provided a mean of 62.6% of all ripe fruits (range: 45–84%) in the habitat. During this period, fig fruit was highly prized: frugivores completely depleted even the largest fig fruit crops (up to ∼80,000 fruits) within one month after the onset of ripening, and often within 1–2 weeks. Thus fig fruits represent a shared, limited resource for the entire frugivore guild. Of the six fig tree species in the study area, we focused on the three most common: *Ficus grevei*, *F. polita* (formerly *F. megapoda*), and *F. reflexa*, which together comprised 81% of all fig tree individuals, and 95% of all fig trees to reach peak fruiting during the study period.

We determined energy intake from fruit consumption via direct, unobtrusive observations on free-ranging frugivores in the field at focal fig trees. We observed foraging by frugivores during peak fruiting stages at each of 34 trees (18 *F. grevei*, seven *F. polita*, and nine *F. reflexa*). At each tree, we conducted observations during peak periods of both diurnal and nocturnal foraging. We used focal observations of individual frugivores at each tree to determine the fruit consumption rate (the rate at which an individual of a frugivore species consumed fig fruit) for each frugivore species at each fig species. At each tree we also used repeated scan censuses of the tree canopy to determine the residency rate (the number of minutes spent in the tree's canopy by all individuals of a species per hour) for each frugivore species at each tree. For each fig species, we used laboratory analysis of collected fruit samples to determine the metabolizable energy available per fruit. We then multiplied the fruit consumption rate (fruits/individual-min), the residency rate (individual-min/hr), and the metabolizable energy per fruit (kJ/fruit) to determine the energy intake rate by each frugivore species at each tree (kJ/hr). We then summed across all fruiting figs in the habitat and converted to energy intake per unit area (kJ/(hr * km^2^)). Further details of these data collection protocols are in Appendix S1.

### Analyses

To test size-energy hypotheses, we determined size-energy relationships with both the indirect and direct methods, and with both the population and size-class approaches, rendering four size-energy scaling relationships. All variables were natural log transformed prior to analysis. For the population approach, population density or population energy intake was regressed onto the body mass of each species. For the size-class approach, we assigned each frugivore species to a size class on the basis of adult body mass (Table 1), and we then summed density or energy intake across all species in the size class. We used normalized-logarithmic binning to assess the form of the size-class relationships. We chose normalized-logarithmic binning because linear binning is known to provide inaccurate estimates of parameters [49]. We did not use maximum likelihood estimation because we expected exponents >−1 and methods for handling these exponents are not commonly available and require estimating a maximum size, which is unknown. Size classes were 0.5 natural log unit bins, normalized for width of the bins (following [49]). Size-class density or size-class energy intake was then regressed onto the midpoint of each size-class bin.

For each of the four size-energy scaling relationships, we first compared the hypothesized linear relationships (energetic equivalence, energy intake increases with body size, energy intake decreases with body size) with a quadratic model for the hypothesized non-linear relationship (energy intake peaks at an intermediate body size). The relative suitability of the linear and quadratic models given the data was evaluated with a model estimation and selection process using information theory (following [50]). While such a model selection process is suitable for the population approach, the process is an ad hoc method for model selection in the size-class approach (see [51] for discussion of issues related to model selection in size-class models). Conclusions would not have differed had we not used a model selection procedure and instead simply used the full model, due to low support for the quadratic term. Since linear models were always selected via the selection procedure (see Results), we then determined the slope (scaling exponent) of the relationship for the selected models from the log-log plot. Because the sign of the slope of these relationships was not known *a priori* and under some hypotheses was expected to be zero, it was not appropriate to use standardized major axis regression [52]. Therefore, we determined slopes in the conventional manner, with ordinary least squares regression (following [6], [14], [16]). To evaluate correlations between the indirect and direct methods, we compared energy use values for all species or size classes common to each pair of size-energy relationships with Pearson's correlation coefficient. Finally, to understand factors that may affect correlations among the size-energy relationships, we used the mathematical links between scaling exponents (*a*+*b* = *c*; see Introduction) in the population approach to calculate the size-metabolism exponent *b* from the measured size-density exponent *a* and the measured size-energy exponent *c*. We completed all statistical analyses with JMP 8.0 [53] and R 2.11.1 [54] statistical software.

## Results

### Indirect method for estimating size-energy relationships from size-density relationships

**Figure 1:**
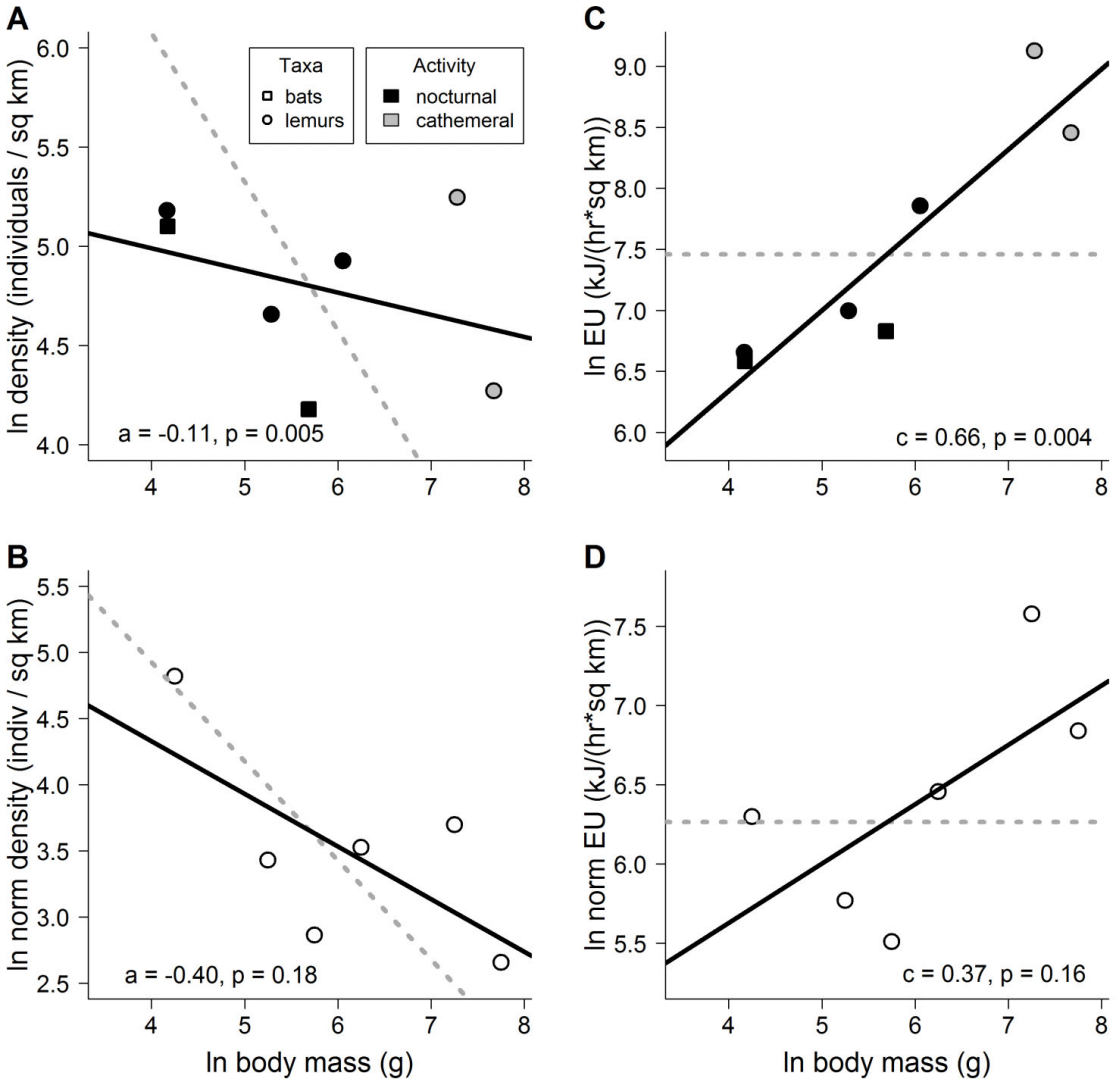
Size-energy relationships, as determined from the indirect method of inference from size-density relationships. (A) The regression slope (solid line) of the size-density relationship with the population approach was significantly shallower (less negative) than the expected slope under the energetic equivalence rule (*a* = −0.75, dotted line). (B) Evidence for a difference from the energetic equivalence rule was weaker in the size-density relationship with the size-class approach. (C) When calculated with the indirect method, the size-energy relationship (where the size-energy scaling exponent, *c*, was equal to 0.66) with the population approach was significantly more positive than expected under energetic equivalence (*c* = 0, dotted line). (D) Evidence was weaker for a difference from energetic equivalence for the size-energy relationship when examined with the size-class approach (*c* = 0.37). Note that EU = energy use, and the y-axes in the size-class analyses (B and D) are normalized for bin width.

We detected 389 frugivorous lemurs in transects. Overall, frugivorous mammal densities ranged from 65.2 individuals per km^2^ in *E. dupreanum* to 189.7 individuals per km^2^ in *E. coronatus* (Appendix S2). Based on these data, the linear models for the size-energy relationship with the indirect method received more support than the quadratic models (Table 2). The slope of the size-density relationship using the population approach (*a* = −0.11, F_1,5_ = 23.23, p = 0.005; Fig. 1A) was shallower (less negative) than expected under the energetic equivalence rule (where *a* is hypothesized to equal −0.75). Evidence for a difference from the energetic equivalence rule was weaker in the size-density relationship, however, when this relationship was examined with the size-class approach (*a* = −0.40, F_1,4_ = 2.60, p = 0.18; Fig. 1B). The slope of the size-energy relationship calculated with the indirect method was greater than expected under energetic equivalence (where *c* is hypothesized to equal 0) with the population approach (*c* = 0.66, F_1,5_ = 24.85, p = 0.004; Fig. 1C), suggesting that results from the indirect method support the hypothesis that population energy use increases linearly with body size. Evidence for such a difference from energetic equivalence in the size-energy relationship with the indirect method, however, was weaker when this relationship was examined with the size-class approach (*c* = 0.37, F_1,4_ = 2.91, p = 0.16; Fig. 1D).

**Table 2:** Comparison of linear and quadratic models for log-log regressions of size-energy relationships, examined with both the indirect and direct methods and both the population and size-class approaches.

Method	Approach	Model Type	AICc	Δ*_i_*	*w_i_*
*Indirect*	*Population*	Linear	20.21	0	0.999
		Quadratic	33.46	13.26	0.001
	*Size class*	Linear	27.12	0	1.000
		Quadratic	55.18	28.06	0.000
*Direct*	*Population*	Linear	63.26	0	0.861
		Quadratic	66.92	3.66	0.139
	*Size class*	Linear	37.19	0	0.999
		Quadratic	50.65	13.46	0.001

Best models were selected on the basis of the small sample size corrected Akaike's Information Criterion (AICc); Δ*_i_* is the difference in AICc between the best and the alternate model, and *w_i_* is the Akaike weight, the weight of evidence for each model given the data (where 1.000 represents the highest likelihood of the model relative to the alternate model).

### Direct method for measuring size-energy relationships

**Figure 2:**
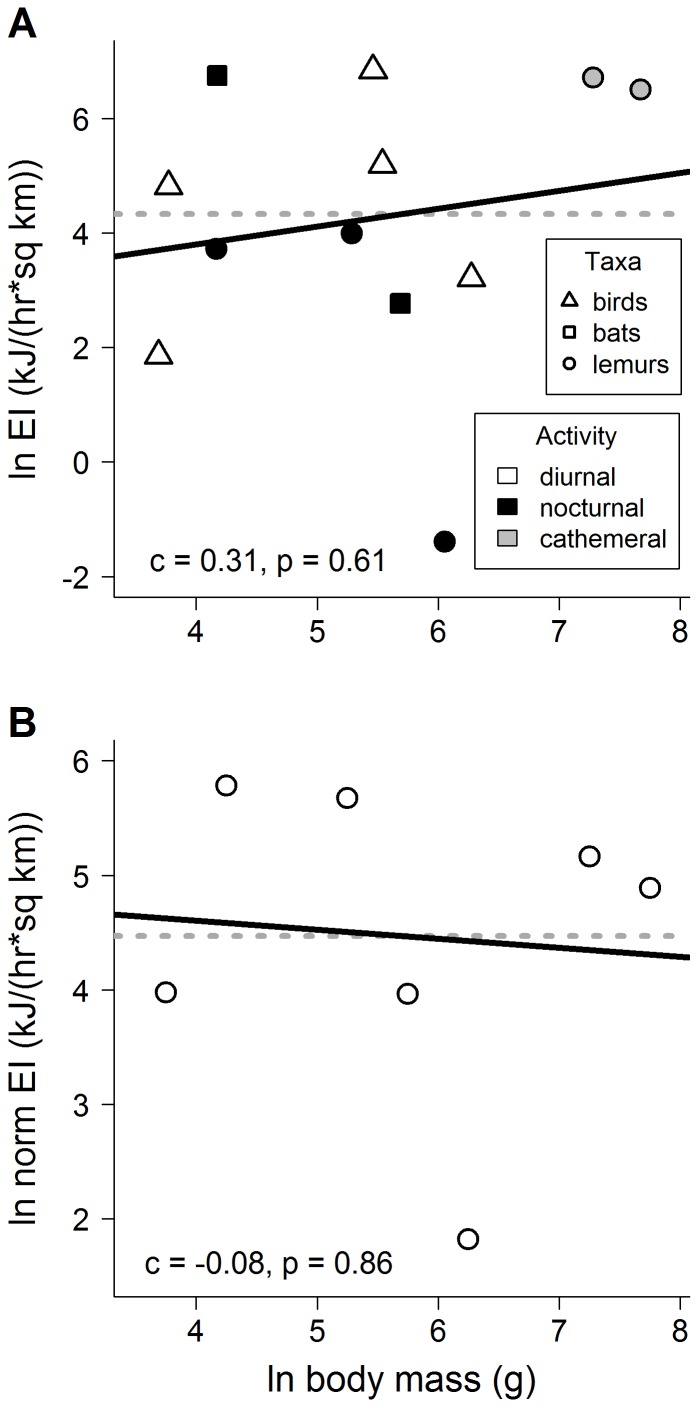
Size-energy relationships, as determined from the direct method of measuring energy intake. Regression slopes (solid lines) of the size-energy relationship with the (A) population approach (where the size-energy scaling exponent, *c*, was equal to 0.31), and (B) size-class approach (*c* = −0.08) were both not significantly different from zero, and therefore were both consistent with the expected slope under energetic equivalence (*c* = 0, dotted lines). Slopes were not qualitatively different if potential outliers (i.e., low values in A and B) were removed. Note that EI = energy intake, and the y-axis in the size class analysis (B) is normalized for bin width.

At our 34 focal trees, we conducted 272 person-hours of observations, with 4080 scan sample observations and 241 focal observations of frugivore foraging of at least 30 seconds in duration. We also collected 1159 fruits from nine fig trees for fruit content analysis (Appendix S1). Population energy intake ranged from 0.25 kJ/(hr * km^2^) by *Phaner electromontis* to 937 kJ/(hr * km^2^) by *Treron australis* (Appendix S2). Based on these data, the linear models for the size-energy relationship with the direct method received more support than the quadratic models (Table 2). Using the direct method, the slopes of the size-energy relationship using both the population approach (*c* = 0.31, F_1,10_ = 0.28, p = 0.61; Fig. 2A) and the size-class approach (*c* = −0.08, F_1,5_ = 0.036, p = 0.86; Fig. 2B) were not different from zero, suggesting that results from the direct method were consistent with the energetic equivalence hypothesis.

### Correlations among size-energy relationships and implications for size-metabolism relationships

The indirect and direct methods of estimating size-energy relationships were uncorrelated (population approach, *r* = 0.19, p = 0.68; size-class approach, *r* = 0.07, p = 0.89). Finally, in the population approach, the slopes of our measured size-density relationship (Fig. 1A) and our measured size-energy relationship (Fig. 2A) imply the slope of the size-metabolism relationship *b* is 0.42.

## Discussion

### Size-energy hypotheses—direct method

Size-energy hypotheses promise to clarify complex patterns of resource use in ecological communities and to provide quantitative predictions of species interactions, but such hypotheses have remained controversial. Rigorous empirical evaluation of size-energy hypotheses requires direct measurement of energy use by foragers during competition for shared, limiting resources [2], [19], but until now tests using direct measurements of energy use had not been completed. We conducted the first such direct test of size-energy hypotheses, and our results (Table 2, Fig. 2) were consistent with the central prediction of the energetic equivalence hypothesis, namely that energy use does not vary systematically with body size. These findings correspond with predictions of metabolic theory [2] and models of energy distribution in ecological communities [55], which suggest a general principle for community ecology: that body size does not confer an advantage to populations or size classes in the competition for energy from shared, limiting resources in communities.

### Size-energy hypotheses—comparison of direct and indirect methods

For comparison with previous studies, we also estimated energy use with the conventional indirect method of inference from size-density relationships and assumptions of constant allometric relationships with metabolism. Previous studies of size-energy hypotheses, which have all used this method, have produced variable results, with most community-scale studies indicating that energy use peaks at intermediate body sizes [13], [17], [20], [26], [30] or increases with body size [11], [16], [21], [22], [56]. These results have been invoked to explain a number of broad ecological patterns, including the island rule (the common pattern of dwarfism and gigantism on isolated islands; [17]), Cope's Rule (the common evolutionary cycle in which large-bodied organisms emerge and become extinct more rapidly than small-bodied species; [16]), and the distribution of colony sizes in eusocial species [22].

Our results from the indirect method and population approach indicated that the slope of the size-energy relationship was linear (Table 2) and positive (*c*>0; Fig. 1C), suggesting that populations of animals of large body size dominate others in the competition for energy. These results were similar to many previous community-scale studies [11], [16], [21], [22], [56], yet these results contrasted with our directly-measured results from the same community (Fig. 2). The indirectly-calculated slope with the size-class approach was also linear (Table 2) but did not differ significantly from the expectation of energetic equivalence (Fig. 1D). Finally, in all cases, results from the indirect method were uncorrelated with results from the direct method (all *r*≤0.19, all p≥0.68, see Results).

The discrepancy in results from the direct and indirect methods – at least in the population approach – may occur due to the inherent biases and assumptions of the indirect method. Notably, the direction of the difference (relatively greater energy use at larger body sizes with the indirect method) represents just the kind of positive correlation between body size and energy use that is expected to derive from the use of body size as a component of both the predictor and response variable in the conventional method of determining and analyzing size-energy relationships (see Introduction).

Alternatively, this discrepancy could also reflect the slightly different focus of both methods. Our study focused on long-lived species, and thus the indirect method, due to its reliance on population densities to estimate energy use, may integrate energy use over all resources and over multiple years. In contrast, our direct method targeted key food resources during a critical period of the year for frugivore survival. Mismatches between the two methods could arise if organisms respond differently to temporal variation in food abundance on the basis of body size, such as for example, if small and large organisms differ in ability (e.g., due to different home range sizes or search areas) to access patchy and unpredictable fruit resources during periods of fruit scarcity [57]. Thus, differences in size-energy relationships resulting from the indirect and direct methods may represent size-dependent temporal variation in energy use.

The contrasting results from the two methods suggest size-density relationships are not appropriate proxies for size-energy relationships. This is not to argue against the examination of size-density relationships, which have provided important insights into key patterns of broad significance for ecology and macroecology [5]. A variety of other promising approaches are also emerging to address energy use in communities [28], [29]. However, our results highlight the limitations and challenges of applying density measurements to understand size-energy relationships, and call into question theories for size-energy relationships that have been derived from measured densities rather than energy use. Without further efforts to empirically validate or refute hypothesized relationships between the two variables with independent lines of evidence, the utility of the density-based methods for estimating local energy scaling relationships will remain unclear.

### Processes of energy regulation—comparison of population and size-class assumptions

Different versions of size-energy hypotheses imply alternative assumptions about processes of energy regulation in communities, and analyses of size-density relationships in multi-trophic communities indicate that energy regulation assumptions may strongly influence observed scaling patterns [2], [23], [32]. In size-energy relationships, population and size-class approaches assume that energy use is driven by metabolic processes related to an organism's taxonomic identity or its body size, respectively [2]. We provided the first empirical comparison of these alternative assumptions for size-energy relationships in the same system, through the use of both population and size-class approaches to quantifying scaling relationships. Although size structure was skewed in our guild toward species with smaller body sizes (Table 1), as is typical of many guilds [58], directly-measured results from the population approach (Fig. 2A) and the size-class approach (Fig. 2B) nonetheless were not different, suggesting that our conclusions about size-energy hypotheses are robust to the energy regulation assumption used. Future comparisons of energy-regulation assumptions could benefit from new techniques for the size-class approach that account for intraspecific variation in body size during species sorting into size class bins [59].

### Mechanisms underlying size-energy relationships

Theory suggests that the energy use of populations or size classes is dependent on their density and individual metabolic rate [2], [6]. While results from our measured size-energy relationship are consistent with energetic equivalence (Fig. 2), results from our measured size-density relationship (Fig. 1A, 1B) add to accumulating evidence [5], [21] that the slope of the size-density relationship is shallower (less negative) at local scales than the −0.75 slope expected from global-scale studies [60], metabolic theory [2], and the energetic equivalence rule [6]. The combination of these two sets of results is unexpected because the conventional indirect method assumes the slope of the size-metabolism relationship, *b*, is +0.75, a value which corresponds with global patterns in size-metabolism relationships [1], [6], [24]. However, our combined results can be explained if the slope of the local size-metabolism relationship in our system was shallower (less positive) than expected. Specifically, our results for the population approach imply a slope of the local size-metabolism relationship of +0.42 (see Results). Local-scale patterns of size-metabolism relationships for interacting species within a community have not previously been explored, but such differences in local-scale variation from global-scale patterns could be common, since metabolic rate is widely variable among taxa [25] and with activity level [61]. In our system the shallower-than-expected slope of the size-metabolism relationship may result from the relatively slow metabolism of frugivores and arboreal species [62], [63] or the energetic constraints imposed by food-scarcity at the end of the dry season [48], [62].

### Challenges in testing energetic equivalence

In addition to the methodological and analytical challenges discussed above, several other factors may complicate tests of scaling relationships in general and the energetic equivalence hypothesis in particular. First, statistical methods for scaling relationships have been controversial, with particular debate over means to reduce bias in estimation of scaling exponents (e.g, [12], [64]). Statistical testing of the energetic equivalence hypothesis poses an additional challenge, as the hypothesis posits a slope of the relationship between body size and energy use of zero, yet regression, the statistical test used to evaluate such relationships, is not designed to enable detection of a zero slope but rather to distinguish deviations from zero [65]. Energetic equivalence is treated statistically as a null hypothesis, and although non-significant results such as those we obtained for the direct measurement of energy use (Fig. 2) have often been taken as positive evidence of the invariance of energy use with body size (e.g., [14], [15]), they cannot be distinguished statistically from a failure to detect a difference from zero [27]. Further application of statistical methods to address these issues is needed [27], [64].

Second, tests of energetic equivalence often exhibit substantial variation around mean trends (e.g., [15]), and both simulation models [66] and empirical evidence [67] predict higher variation at smaller body size ranges. Such variation may decrease power and make it harder to detect scaling relationships empirically in community-scale studies [27]. Greater power could be obtained by examining a greater number of species or a greater body mass range, but this will not be possible to accomplish while controlling for resource use in many communities such as ours, where species numbers and body mass ranges in a guild are limited (Table 1; [66]).

In our study, data were highly variable for all relationships (Figs. 1, 2), but variance around the mean scaling trend was much greater for the direct method (standard deviation >2 natural log units; Fig. 2A, 2B) than for the indirect method (st. dev. <0.5 natural log units; Figs. 1C, 1D). This discrepancy in variances in the two methods may be due to the use of an assumed constant metabolic scaling exponent, *b*, of +0.75 for all species to calculate energy use in the conventional indirect method (see Introduction), despite the highly variable nature of this exponent by taxon [25] or community (as noted above, *b* differed from this assumption for our system). By ignoring such community-scale variation in size-metabolism relationships, the conventional indirect approach may obscure an important source of error and greatly overestimate the precision of reported size-energy relationships. Any future analyses using the indirect method should explicitly account for this expected variance in the metabolic scaling exponent.

While size-energy relationships offer an explanation for the mean scaling trend among species in a community, they do not provide an explanation for the high levels of residual variation in the data. Such variation may be due to differences in a number of evolutionary and ecological factors, including relatedness, niche differences, dispersal patterns, predation risk, or trade-offs among growth, mortality, fecundity and other life history variables [28], [29], [66], [68]. Understanding the relative influence of body size and each of these other factors on energy use remains an important question in community ecology [29]. Further insights can be gained through studies comparing the relative influence of each variable on energy use, and through additional data collected across communities and over time [27], [28].

### Conclusions

Investigations of local scaling relationships enable prediction of community patterns of population density and resource use; provide important links among individual-, population-, and community-level processes in ecology; and clarify mechanisms underlying resource partition in ecological communities [5]. By providing the first direct measurement of the size-energy relationship, and by controlling for factors that have often confounded previous studies, our study enabled new insights into the form of the local size-energy relationship. Specifically, our study is consistent with the idea that energy use is independent of body size. Our results were robust to the energy regulation assumption used, and suggest that shallower-than-expected relationships of body size to density and metabolism may combine to produce energetic equivalence at local scales. Our study focused on foragers on shared, limiting resources in communities because competition for resources is the proposed mechanism underlying energetic equivalence, but such results can be extended to multi-trophic communities [2], [23], [32], [64], [69], [70].

In addition to directly evaluating the size-energy relationship, our study compared alternative methods to understand this relationship at the community scale. Our direct method of measurement of the size-energy relationship contrasts sharply with interpretations that would have been derived from the conventional indirect method, suggesting that the size-density relationship in a community cannot be used to infer the size-energy relationship. Further, the differences we observed in size-density relationships between our local study and past global studies highlight that body size relationships may describe distinct phenomena, be generated by differing mechanisms, and exhibit contrasting patterns at different spatial scales.

More broadly, our focus in this study was on evaluating size-energy hypotheses, and our finding that energy intake was independent of body size (Fig. 2) was consistent with the energetic equivalence hypothesis. This finding is of particular interest for ecological theory, since energetic equivalence implies that small and large animals will produce similar amounts of biomass over time [6]; population growth and carrying capacities will be related predictably to body size [8]; resources will be used at similar rates by groups of organisms of different sizes [2]; and community assembly will not be driven by or oriented around an optimal body size [71]. Our findings therefore have important implications for predicting species interactions and for understanding many aspects of community structure and dynamics.

## Supporting Information

Appendix S1 **Methods for direct measurement of energy intake by frugivores.**
(PDF)

Appendix S2 **Measured values for density and population energy use by species.**
(PDF)
