# Supplementary material for: Size-Energy Relationships in Ecological Communities
Source: PLoS One. 2013 Aug 7;8(8):e68657. doi: 10.1371/journal.pone.0068657 (PMC3737256; doi:10.1371/journal.pone.0068657)
Supplement: Appendix S1 — Methods for direct measurement of energy intake by frugivores. (PDF) [file pone.0068657.s001.pdf]

Appendix S1. Methods for direct measurement of energy intake by frugivores.

Fig tracking

Since fruit ripening in Ankarana fig species was asynchronous at the population scale, we tracked the reproductive status of all mature fig trees throughout the study area. Overall, we located and tracked the reproductive status of 149 individuals of the three focal fig tree species (54 *F. grevei*, 54 *F. polita*, and 41 *F. reflexa*). Of these, 40 trees (20 *F. grevei*, seven *F. polita* and 13 *F. reflexa*) reached fruiting peaks during the study period. We observed frugivore foraging at 34 of these trees (18 *F. grevei*, seven *F. polita*, and nine *F. reflexa*).

Focal tree observations and observations of foraging by frugivores

We conducted frugivore observations during the stage of the tree's fruiting period with peak frugivore visitation, as determined by a rapid increase in observed frugivore activity in the canopy and in collection of fig feeding refuse (fallen fruit, marked fruit, partially eaten fruit, and ejecta pellets and feces dominated by fig material) in tarps below the trees' canopies. Fruiting peaks typically lasted for three days at *F. grevei* and *F. polita* and for seven days at *F. reflexa*. We conducted two observations at each fig tree during successive diurnal and nocturnal periods of high frugivore activity, just before (15:30 - 18:30) and after (18:30 - 21:00) sunset. The timing of these observation periods enabled us to observe the tree when most foraging occurred (during daily foraging peaks), and to conduct diurnal and nocturnal observations when resource availability was most similar (during successive periods). Feeding refuse collected over 24 hr in fruit traps placed beneath each tree indicated that relative activity rates for birds, lemurs, and bats at trees during observation periods were representative of overall rates.

From a ground-based vantage, we directly observed frugivore foraging activity with binoculars and night vision equipment with third generation image intensification [1] of either passive ambient light or reflected light from an infrared illuminator. We also occasionally used brief spotlighting to reinforce direct nocturnal observations or to distinguish species; the relatively dim light arriving at the tree did not visibly affect foragers.

Diurnal and nocturnal observation periods were each two hours long and performed by two observers who alternated roles each half hour. Data collection was as follows for all species except *Rousettus madagascariensis* (see below for methods for this species). The first observer conducted focal observations on individual frugivores, distributing observations among all frugivore species present without repeating observations on any individual. This observer counted fruits consumed by the focal individual and recorded the duration of the observation to determine the fruit consumption rate (fruits / individual-min), the rate at which an individual of a frugivore species consumed fig fruit. Observations continued whether the individual was feeding or not, and ended when the focal individual left the tree or moved out of sight, or when 15 minutes had elapsed. Observations of less than 30 seconds were excluded from analyses.

The second observer conducted scan sample observations, counting the number of individuals of each frugivore species present in the focal tree's canopy, via point observations at two-minute intervals. We summed counts for each species across the 60 intervals to determine the residency rate (individual-min / hr), the number of minutes spent in the tree's canopy by all individuals of a species per hour. We standardized residency rates on the basis of the proportion of the tree visible from the vantage point.

Observations of foraging by Rousettus madagascariensis

Different foraging behavior by the fruit bat *R. madagascariensis* necessitated some different observation and calculation methods. Whereas all other Ankarana frugivores consumed fruit while present in a tree's canopy, *R. madagascariensis* removed fruit in flight. This behavior is similar to that of other *Rousettus* species, which may hover at a branch and carry fruit and consume it at nearby feeding sites [e.g., 2,3]. Thus, during nocturnal observations, the second observer also kept a continual watch for *R. madagascariensis* visits to the tree, recording any direct visits to branches. Fly-bys and flights around the tree were not counted. All direct visits by *R. madagascariensis* during the observation period were tallied (not solely those that occurred at the two-minute interval points) to determine the visitation rate (visits / hr). We standardized visitation rate on the basis of the proportion of the tree visible from the vantage point.

We also determined fruit removal success (fruits / visit) on the basis of *R. madagascariensis* foraging behavior at fig trees. Given the precision of *R. madagascariensis* visits to branches, fruit removal was not greater than one per visit, but due to the speed of *R. madagascariensis* visits, we were unable to accurately measure whether a fruit was successfully removed during each visit. However, the bats typically flew around the tree crown prior to visiting a branch, then directly away immediately thereafter; second attempts were uncommon. Hence, we calculated *R. madagascariensis* consumption under two assumptions of fruit removal success, where fruit removal success = 0.5 or 1.0 fruits per visit to a branch. Only the results using the former assumption are presented here, as the two assumptions had no qualitative effect on results or conclusions about size-energy hypotheses.

Laboratory analysis and calculations of metabolizable energy in fig (Ficus) fruits

To determine the energy content of fruit from each fig species, we first collected fig fruit from each species, and measured wet mass of fruit samples in the field. We then convection dried [4] samples to constant mass (1-3 days); moisture content was the difference between wet and dry mass. We used laboratory analysis of dried fruit samples to determine protein, fat, and carbohydrate content with standard methods [5; A&L Western Agricultural Laboratories, Modesto, CA, USA], and used moisture content to convert components of fruit to proportions of wet mass. Analyses were completed on whole fruits, since differences in calculated energy content between whole-fruit assays and pulp-only assays in figs are small and consistent between fig species [6]. Next, we calculated metabolizable energy content by multiplying each nutrient by standard conversion factors for energy in fig fruits: 14.07 kJ / g in protein, 35.04 kJ / g in fat, and 15.07 kJ / g in carbohydrate [7]. We then summed the energy from each nutrient, and multiplied by mean fruit biomass to estimate the total metabolizable energy available per fruit (kJ / fruit) of each fig species (Table S1).

Energy intake rates

We determined energy intake rate (kJ / hr), as the mean rate of energy consumption by each frugivore species at each tree species. This rate was calculated, for all species except *R. madagascariensis*, as the product of the fruit consumption rate, the standardized residency rate, and the metabolizable energy per fruit. For *R. madagascariensis*, this rate was calculated as the product of the standardized visitation rate, the fruit removal success, and the metabolizable energy per fruit.

To determine energy use per unit area (kJ / (hr * km^2)), we averaged the overall energy consumption rate by each frugivore species at each tree, and weighted by the relative density of fruiting trees of each fig species in the study site. The weighting was determined as the product of fruiting fig density (the number of trees of each fig species reaching their peak fruiting stage during the study period per unit area, measured in trees / km^2), fruiting duration (the number of days a tree contained ripe abundant fruit, measured in days / tree), and tracking effort (the number of days we tracked fig trees during the study period, measured in $days^{-1}$).

Table S1. Fruit content in ripe fruits of three fig (*Ficus*) species.

**Trees**	**Fruit content**					**Metabolizable energy**	
species	wet mass (g)[a]	moisture (%)[b]	protein (%)[b]	fat (%)[b]	carbohydrate (%)[b]	energy / mass (kJ / g)[c]	energy / fruit (kJ)
F. grevei	1.72	78.6	1.67	0.926	16.9	3.11	5.34
F. polita	7.12	80.7	1.72	1.52	14.4	2.94	21.0
F. reflexa	0.54	74.2	2.05	1.69	19.5	3.82	2.06

[a] Mean of 327 *F. grevei* fruits, 224 *F. polita* fruits, and 608 *F. reflexa* fruits at three trees of each species.

[b] Measures of fruit content are shown as a percentage of wet mass. Analysis of 101 *F. grevei* fruits, 102 *F. polita* fruits, and 102 *F. reflexa* fruits at one tree of each species.

[c] See text in section entitled "*Laboratory analysis and calculations of metabolizable energy in fig (Ficus) fruits*" above in this Appendix for procedure for calculating metabolizable energy from fruit nutritional content.

References

1. Allison NL, DeStefano S (2006) Equipment and techniques for nocturnal wildlife studies. Wildlife Society Bulletin 34: 1036-1044.

2. Izhaki I, Korine C, Arad Z (1995) The effect of bat (*Rousettus aegyptiacus*) dispersal on seed-germination in eastern Mediterranean habitats. Oecologia 101: 335-342.

3. Sewall BJ, Granek EF, Trewhella WJ (2003) The endemic Comoros islands fruit bat *Rousettus obliviosus*: ecology, conservation, and Red List status. Oryx 37: 344-352.

4. Liesner R (1995) Field Techniques Used by Missouri Botanical Garden. St. Louis: Missouri Botanical Garden.

5. Cunniff P (1995) Official Methods of Analysis of AOAC International, 16th edition. Arlington, Virginia: AOAC International.

6. Wrangham RW, Conklin NL, Etot G, Obua J, Hunt KD, et al. (1993) The value of figs to chimpanzees. International Journal of Primatology 14: 243-256.

7. U.S. Department of Agriculture (2008) USDA National Nutrient Database for Standard Reference, Release 21. Agricultural Research Service, U.S. Department of Agriculture. Nutrient Data Laboratory. http://www.ars.usda.gov/ba/bhnrc/ndl.
