# Supplementary material for: Size-Energy Relationships in Ecological Communities
Source: PLoS One. 2013 Aug 7;8(8):e68657. doi: 10.1371/journal.pone.0068657 (PMC3737256; doi:10.1371/journal.pone.0068657)
Supplement: Appendix S2 — Measured values for density and population energy use by species. (PDF) [file pone.0068657.s002.pdf]

Appendix S2. Measured values for density and population energy use by species.

Table S2. Measured densities for mammals, and population energy use (PEU) by direct method for all species

**Species**	**English name**	**Taxon**	**Density (individuals / km^2)**	**PEU, direct method (kJ / (hr * km ^{2}))**
Saroglossa aurata	Madagascar Starling	Bird	-	6.43
Hypsipetes madagascariensis	Madagascar Bulbul	Bird	-	124
Microcebus tavaratra	northern rufous mouse lemur	Lemur	177.7	41.4
Rousettus madagascariensis	Madagascar rousette	Bat	164.1	853
Cheirogaleus medius	fat-tailed dwarf lemur	Lemur	105.3	54.1
Treron australis	Madagascar Green Pigeon	Bird	-	937
Coracopsis nigra	Lesser Vasa Parrot	Bird	-	180
Eidolon dupreanum	Madagascar straw-colored fruit bat	Bat	65.2	16.0
Phaner electromontis	Amber Mountain fork-marked lemur	Lemur	137.8	0.25
Coracopsis vasa	Greater Vasa Parrot	Bird	-	24.8
Eulemur coronatus	crowned lemur	Lemur	189.7	823
Eulemur sanfordi	Sanford’s brown lemur	Lemur	71.6	667
